# Data analysis with Shapley values for automatic subject selection in Alzheimer’s disease data sets using interpretable machine learning

**DOI:** 10.1186/s13195-021-00879-4

**Published:** 2021-09-15

**Authors:** Louise Bloch, Christoph M. Friedrich, for the Alzheimer’s Disease Neuroimaging Initiative

**Affiliations:** 1 Department of Computer Science, University of Applied Sciences and Arts Dortmund, Dortmund, 44227 Germany; 2 Institute for Medical Informatics, Biometry and Epidemiology (IMIBE), University Hospital Essen, Essen, 45122 Germany

**Keywords:** Machine learning, Data Shapley, Interpretability, AIBL, ADNI, Shapley values, Mild cognitive impairment, Alzheimer’s disease

## Abstract

**Background:**

For the recruitment and monitoring of subjects for therapy studies, it is important to predict whether mild cognitive impaired (MCI) subjects will prospectively develop Alzheimer’s disease (AD). Machine learning (ML) is suitable to improve early AD prediction. The etiology of AD is heterogeneous, which leads to high variability in disease patterns. Further variability originates from multicentric study designs, varying acquisition protocols, and errors in the preprocessing of magnetic resonance imaging (MRI) scans. The high variability makes the differentiation between signal and noise difficult and may lead to overfitting. This article examines whether an automatic and fair data valuation method based on Shapley values can identify the most informative subjects to improve ML classification.

**Methods:**

An ML workflow was developed and trained for a subset of the Alzheimer’s Disease Neuroimaging Initiative (ADNI) cohort. The validation was executed for an independent ADNI test set and for the Australian Imaging, Biomarker and Lifestyle Flagship Study of Ageing (AIBL) cohort. The workflow included volumetric MRI feature extraction, feature selection, sample selection using Data Shapley, random forest (RF), and eXtreme Gradient Boosting (XGBoost) for model training as well as Kernel SHapley Additive exPlanations (SHAP) values for model interpretation.

**Results:**

The RF models, which excluded 134 of the 467 training subjects based on their RF Data Shapley values, outperformed the base models that reached a mean accuracy of 62.64% by 5.76% (3.61 percentage points) for the independent ADNI test set. The XGBoost base models reached a mean accuracy of 60.00% for the AIBL data set. The exclusion of those 133 subjects with the smallest RF Data Shapley values could improve the classification accuracy by 2.98% (1.79 percentage points). The cutoff values were calculated using an independent validation set.

**Conclusion:**

The Data Shapley method was able to improve the mean accuracies for the test sets. The most informative subjects were associated with the number of ApolipoproteinE *ε*4 (ApoE *ε*4) alleles, cognitive test results, and volumetric MRI measurements.

## Background

Alzheimer’s disease (AD) is a neurodegenerative disease [1] and the most frequent cause of dementia [2]. In 2018, there were approximately 50 million patients [2] with dementia worldwide. This number is expected, to increase up to 152 million by 2050 [2]. Two thirds of those patients suffer from AD [2]. At the moment, there is no causal therapy to cure AD [3].

The early identification of patients at risk to develop AD, and the development of preclinical markers, is important to recruit subjects for therapy studies that aim to stop the progression among the AD continuum [4]. On this continuum, individuals that develop cognitive impairment not inferring with everyday activities are considered as having mild cognitive impairment (MCI) due to AD. Subjects with MCI have a higher risk to develop AD [5] than cognitively healthy individuals. However, not all individuals with evidence of AD brain changes will prospectively develop symptoms of MCI or dementia. Thus, the differentiation between progressive MCI (pMCI) subjects who will prospectively develop AD and subjects with a stable course of MCI (sMCI) is important [6].

Machine learning (ML) was successfully applied to AD detection in various studies [7–9]. However, AD is a heterogeneous disease [10, 11], which leads to diverse disease patterns in ML data sets. Multicentric study designs, varying magnetic resonance imaging (MRI) acquisition protocols, and inaccuracies in MRI processing increase the data variability. The variability in MRI processing is, for example, caused by MRI segmentation errors [12]. Due to the high data variability, it is often hard for ML methods to distinguish between disease variability and noise, which increases the risk of overfitting [13]. An overfitted ML model achieves good classification results for the training set but worse results for independent test data [13]. Overfitted models thus do not focus on the most relevant distinction criteria but were potentially confused by noisy data. The motivation of this research is, to prevent the overfitting of ML models and thus increase generalization. One idea to overcome this problem is to focus the training data set on highly representative subjects. It is expected that this focus will decrease the accuracies for the training set but will increase them for independent test sets [14]. Respectively, the model becomes more generalizable and less susceptible to noise.

The identification of the subjects with the most informative data was implemented using Data Shapley [15]. This method valuated the quality of a subject by its contribution to ML models.

### Related work

Outlier detection [16] is a common strategy in ML preprocessing, improving the classification results and robustness of ML models [17]. However, there are multiple definitions of outliers in this context. Classical outlier detection methods [16], like isolation forest [18], density-based spatial clustering of applications with noise (DBSCAN) [19], local outlier factor (LOF) [20], generative adversarial network (GAN)-based [21] outlier detection [22], and self-supervised outlier detection (SSD) [23], define samples strongly different from the remaining data set as outliers [24]. An unsupervised fuzzy c-means clustering to identify outlier subjects during AD detection was proposed in Duraisamy et al. [25]. Based on the reduced data set, a weighted probabilistic neural network [26] was trained. The data set included texture and shape MRI features extracted from the hippocampus and posterior cingulate cortex. The approach was validated for 509 subjects (137 AD, 210 MCI, 162 cognitive normals (CN)) of the Alzheimer’s Disease Neuroimaging Initiative (ADNI) [27], and 74 subjects (21 CN, 37 MCI, 16 AD) from the Bordeaux-3-city data set [28]. The results showed accuracies of 98.63% (CN vs. AD), 95.4% (CN vs. MCI), and 96.4% (MCI vs. AD) for the ADNI data set. The exclusion of outlier subjects improved the classification results.

For the detection of both subjects with noisy data and less important features, a semi-supervised linear discriminant analysis was developed in Adeli-Mosabbeb et al. [29]. The algorithm was evaluated for two synthetic data sets and two real-world data sets for the detection of Parkinson’s disease and AD. For AD detection, 93 AD, 202 MCI, and 101 CN ADNI subjects were included. The grey matter (GM) volumes of predefined MRI regions of interest (ROIs) and the mean intensities of fluorodeoxyglucose (FDG) positron emission tomography (PET) scans were used as features. The results outperformed comparable models by reaching accuracies of 91.8% (CN vs. AD) and 89.8% (CN vs. MCI).

A framework that enables both feature and sample selection based on a hierarchical approach was introduced in An et al. [30]. The approach was validated for a subset containing 737 ADNI-1 subjects (204 CN, 205 sMCI, 157 pMCI, 171 AD). GM volumes extracted from the MRI scans and single-nucleotide polymorphisms (SNPs) were used as features for the experiments. A linear support vector machine (SVM) [31] accomplished the final classification. The cross-validation results outperformed multiple feature selection methods by reaching accuracies of 92.4% (CN vs. AD), 80.1% (CN vs. MCI), and 80.8% (sMCI vs. pMCI).

Confident learning [32] is related to outlier detection but with a different definition of outliers. The main idea in confident learning is to automatically identify samples with incorrect or noisy labels in ML data sets. A model-agnostic confident learning approach, estimating the joint distribution between noisy and corrected labels, was implemented in Northcutt et al. [32]. The identification of noisy labels depends on the out-of-sample predicted probabilities of ML models. The exclusion of images with noisy labels from the ImageNet [33] data set using a ResNet18 [34] convolutional neural network (CNN) [35] led to improved results.

Another definition of outliers called Instances that Should be Misclassified (ISM) was introduced in Smith and Martinez [14]. This paper also provided an outlier detection method called PReprocessing Instances that Should be Misclassified (PRISM). Samples that do not lead to improved ML models but overfitting are identified as ISMs. The experiments also showed improved results for 53 classification data sets selected from the University of California, Irvine (UCI) ML repository (https://archive.ics.uci.edu/ml/index.php, Accessed: 18 May 2021), training nine ML models based on the reduced data sets.

A similar approach, called Data Shapley, was introduced in Ghorbani et al. [15]. This approach valuates data samples based on Shapley values [36]. Shapley values are affiliated with coalition game theory. The aim is to fairly calculate the coalition of each data sample to the collaborative classification result. The fairness of Shapley values is achieved by considering the coalition in each subset of samples.

The idea of Data Shapley is to fairly valuate the samples in a data set based on their contribution to the overall model performance. This approach was successfully applied for pneumonia detection in Tang et al. [37] for the chest X-ray [38] data set, resulting in improved classification results.

This article transferred the method described in Tang et al. [37] to early AD detection. The aim of this work is to prevent overfitting in heterogeneous AD data sets and thus train more robust ML models. Additionally, subjects that were classified as being less representative were identified and examined. In this context, Shapley values were also used to explain black-box models similar to previous work [39].

## Material and methods

**Fig. 1:**
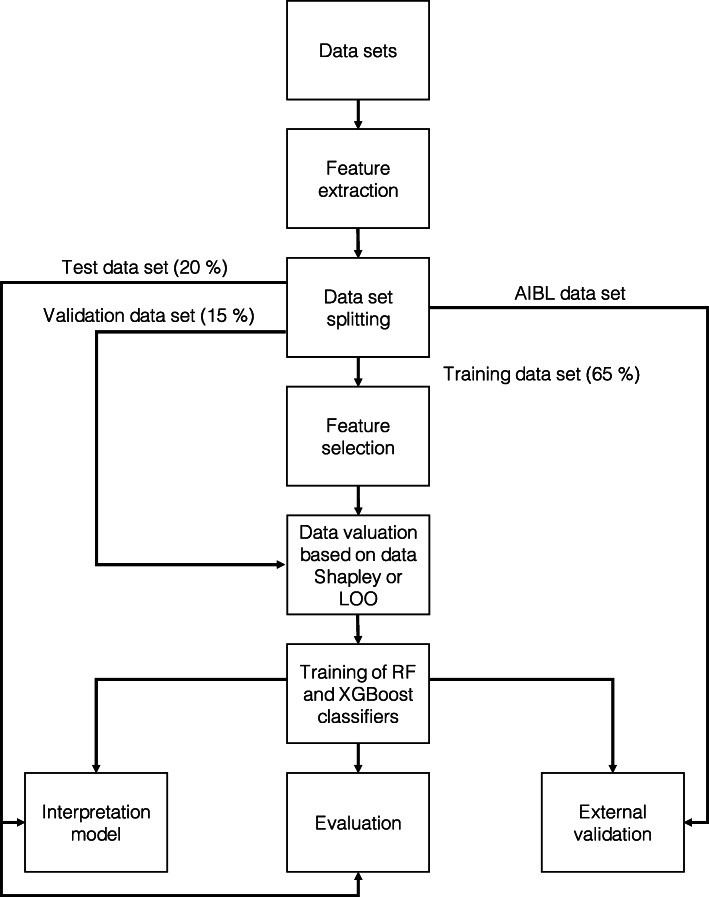
Implemented ML workflow. The experiments were based on data from ADNI and AIBL. Volumetric features were extracted for one BL MRI scan per subject. The ADNI data set was randomly split into a 65% training, 15% validation, and 20% test set. RF feature selection was implemented to extract the most important MRI features for the training set. Those MRI features were concatenated with demographic features and cognitive test scores. Data valuation with Data Shapley values was implemented to detect the subjects with the most informative data. Black-box RF and XGBoost models were trained and validated. Shapley values were calculated for black-box model interpretation

This section describes the material and methods of the ML workflow visualized in Fig. 1. The ML workflow was implemented using the programming language Python version 3.6.9 [40]. The “Hyperparameters of implementation” section summarizes the parameters of the implementation. First, data sets and subject preselection were defined. Afterwards, volumetric features were extracted from MRI scans. ADNI data were split on the subject level into 65% training, 15% validation, and 20% test sets. Data valuation using Data Shapley was executed to identify those subjects with the most informative data. Leave-one-out (LOO) [41] data valuation was executed as a comparison method. On the training set, random forest (RF) [42], and eXtreme Gradient Boosting (XGBoost) [43] models, were trained. Finally, Kernel SHapley Additive exPlanations (SHAP) values [44] were calculated to interpret the models.

### Data sets

In this article, data from two AD cohorts were included. The models were trained and validated using data from the ADNI [27] cohort. External validation was performed using data from the Australian Imaging, Biomarker and Lifestyle Flagship Study of Ageing (AIBL) [45].

ADNI (https://adni.loni.usc.edu, Accessed: 18 May 2021) was launched in 2003 as a public-private partnership. The primary goal of ADNI is to test whether a combination of biomarkers can measure the progression of MCI and AD. Those biomarkers include serial MRI, PET, and biological markers, as well as clinical and neuropsychological assessments. The ADNI cohort recruited subjects from more than 60 sites in the USA and Canada and consists of four phases (ADNI-1, ADNI-2, ADNIGO, and ADNI-3). The subjects were assigned to three diagnostic groups. CNs have no problems with memory loss. Subjects with AD meet the criteria for probable AD defined by the National Institute of Neurological and Communicative Disorders and Stroke–Alzheimer’s Disease and Related Disorders Association (NINCDS-ADRDA) [46]. The diagnostic criteria of ADNI were explained in more detail in Petersen et al. [27]. The data set was downloaded on 27 Jul 2020 and included 2250 subjects.

AIBL (https://aibl.csiro.au/, Accessed: 18 May 2021) is the largest AD study in Australia and was launched in 2006. AIBL aims to discover biomarkers, cognitive test results, and lifestyle factors associated with AD. As AIBL focuses on early AD stages, most of the subjects are CN. The MCI subjects of AIBL met the criteria described in Winblad et al. [47], whereas AD diagnoses follow the NINCDS-ADRDA criteria [46] for probable AD. The diagnostic criteria of AIBL were described in Ellis et al. [45]. Approximately half of the CN subjects recruited in AIBL show memory complaints [45]. AIBL data version 3.3.0 was downloaded on 19 Sep 2019 and included 826 subjects.

**Fig. 2:**
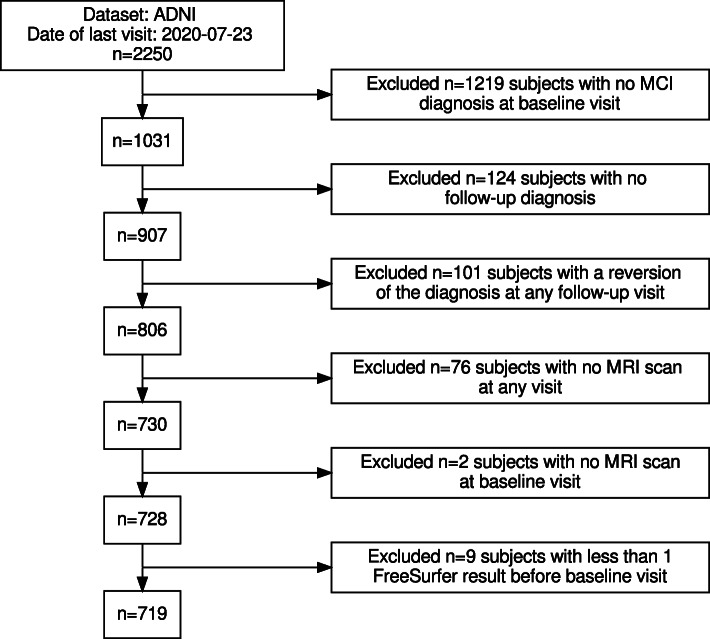
Flow chart of the subject selection for the ADNI data set. On the right side of this diagram, exclusion criteria and the number of subjects excluded for each criterion are described. The number of remaining subjects is summarized on the left side of this diagram. Finally, 719 subjects were included in the experiments

The proposed workflow aims to predict whether subjects with a baseline (BL) diagnosis of MCI will prospectively convert to AD. The data set was not limited to a conversion period to include as many subjects from the original data set as possible. This selection makes the data set more diverse. The sMCI group included subjects with MCI as BL diagnosis and no diagnostic changes in all subsequent visits. Subjects with no follow-up diagnosis, and subjects with a reversion to CN at any visit, were excluded. The pMCI diagnostic group included MCI subjects, which converted to a stable diagnosis of AD. Thus, pMCI subjects, which reverted to CN or MCI, were excluded. Those exclusion criteria, and the number of subjects excluded from the ADNI data set by each criterion, are visualized in Fig. 2. The ADNI data set initially included 2250 subjects. 1219 subjects with no MCI diagnosis at the BL visit were excluded. Afterwards, 124 MCI-subjects with no follow-up diagnosis were excluded. The diagnosis of 101 subjects reverted at any follow-up visit. For 76 subjects, no MRI scans were available in the “ADNIMERGE” [48] merged ADNI data set, and two additional subjects had no BL MRI scan available. The image pipeline described in the “Feature extraction” section failed for the BL MRI scans of nine subjects. Overall, 719 subjects—400 sMCI and 319 pMCI—were included in the experiments. Demographic data, the number of ApolipoproteinE *ε*4 (ApoE *ε*4) alleles, the Mini-Mental State Examination (MMSE), and Clinical Dementia Rating (CDR) scores are summarized in Table 1. In the pMCI group, the minimal conversion time was 5.0 months, and the maximum conversion time was 137.7 months. For the sMCI group, the latest diagnosis was recorded between 4.7 and 156.2 months after BL.

**Table 1:** Demographic data, cognitive tests, and the number of ApoE *ε*4 alleles of the selected ADNI subjects separated by diagnosis group

Variable	sMCI	pMCI	*Σ*	*p* value
*n*	400	319	719	
Age (in years)	73.2 ±7.5	74.0 ±7.1	73.6 ±7.3	0.1281
Gender (proportion of females)	40.3%	40.1%	40.2%	1.0000
Gender (proportion of males)	59.8%	59.9%	59.8%	
MMSE	27.8 ±1.8	27.0 ±1.7	27.4 ±1.8	<0.0001
CDR	0.5 ±0.0	0.5 ±0.0	0.5 ±0.0	0.2640
ApoE *ε*4 (proportion of subjects with 0 alleles)	56.8%	34.2%	46.7%	<0.0001
ApoE *ε*4 (proportion of subjects with 1 allele)	34.0%	49.5%	40.9%	
ApoE *ε*4 (proportion of subjects with 2 alleles)	9.3%	16.3%	12.4%	
Time to final diagnosis in months	47.3 ±32.6	30.6 ±24.7	39.8 ±30.4	<0.0001

For continuous features, mean and standard deviation are given. p value are calculated using Mann-Whitney *U* test [49, 50] for continuous features and using *χ*^2^-test for ordinal and nominal features

**Fig. 3:**
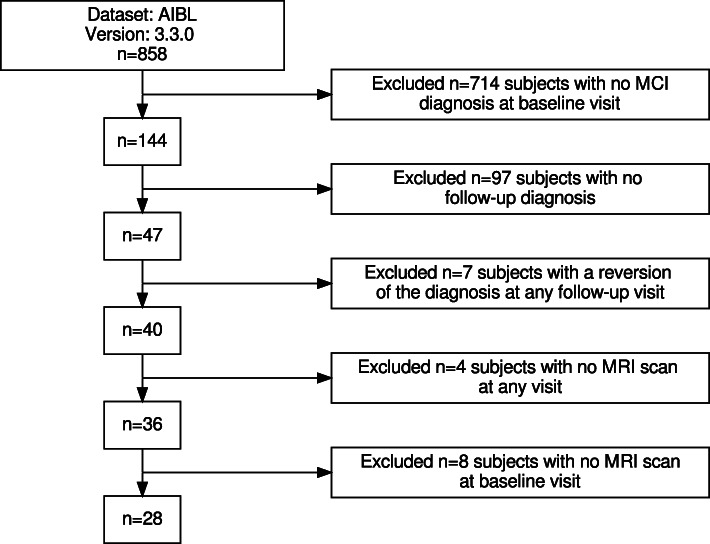
Flow chart of the subject selection for the AIBL data set. On the right side of this diagram, exclusion criteria and the number of subjects excluded for each criterion are described. The number of remaining subjects is summarized on the left side of this diagram. Finally, 28 subjects were included in the experiments

The same exclusion criteria were applied to the AIBL cohort. Figure 3 summarizes the exclusion process for the AIBL data set. Initially, the AIBL data set v3.3.0 contained 858 subjects. 714 of those subjects had a diagnosis of CN or AD. Another 97 subjects had no follow-up diagnosis available. The diagnosis of seven subjects reverted at any follow-up visit. Those subjects were excluded from the data set. Another four subjects had no MRI scan, and eight subjects had no MRI scan available at the BL visit. Those criteria result in 28 AIBL subjects included in these experiments. The demographics, and cognitive test results at the BL visit, are summarized in Table 2.

**Table 2:** Demographic data, cognitive test scores, and the number of ApoE *ε*4 alleles of the selected AIBL subjects separated by diagnosis group

Variable	sMCI	pMCI	*Σ*	*p* value
*n*	16	12	28	
Age (in years)	77.8 ±6.9	75.3 ±5.8	76.8 ±6.5	0.2856
Gender (proportion of females)	37.5%	33.3%	35.7%	1.0000
Gender (proportion of males)	62.5%	66.7%	64.3%	
MMSE	28.0 ±1.7	26.3 ±1.6	27.3 ±1.8	0.0133
CDR	0.4 ±0.2	0.5 ±0.0	0.5 ±0.1	0.2317
ApoE *ε*4 (proportion of subjects with 0 alleles)	56.3%	16.7%	39.3%	0.0550
ApoE *ε*4 (proportion of subjects with 1 allele)	37.5%	50.0%	42.9%	
ApoE *ε*4 (proportion of subjects with 2 alleles)	6.3%	33.3%	17.9%	
Time to final diagnosis in months	41.3 ±22.4	28.4 ±14.2	35.7 ±20.1	<0.2457

For continuous features, mean and standard deviation are given. *p* values are calculated using Mann-Whitney *U* test [49, 50] for continuous features and using *χ*^2^-test for ordinal and nominal features

### Feature extraction

The acquisition protocols for the ADNI scans were described in detail in Jack et al. [51] for ADNI-1, in Jack et al. [52] for ADNI-2 and ADNIGO, and in Weiner et al. [53] for ADNI-3. This research included T1-weighted MRI scans recorded at the BL visit. The acquisition parameters differ between scanners. During the ADNI-1 study phase, scans were recorded using a field strength of 1.5 T. In the remaining study phases, 3.0 T was used as the MRI field strength.

The AIBL T1-weighted MRI scans followed the protocol of the ADNI 3D T1-weighted sequences. All scans had a resolution of 1×1×1.2 mm.

Using the FreeSurfer v6.0 [54] recon-all pipeline, volumetric features were extracted from 34 cortical areas per hemisphere of the Desikan–Killiany–Tourville (DKT) atlas [55], 34 subcortical areas [56], and the estimated Total Intracranial Volume (eTIV). FreeSurfer shows good test-retest reliability between scanners and across field strengths [57]. The resulting 103 volumetric features were normalized by eTIV as recommended for volumes in Westman et al. [58].

### Data set splitting

At the subject level, the ADNI data set was split into three distinct subsets. The training set included 65% (467 subjects) of the data, the validation set included 15% (108 subjects), and the test set consisted of the remaining 20% (144 subjects). The splitting was executed within each diagnostic group to ensure representative distributions. As an additional external test set, the AIBL data set was used. During model training, none of the AIBL subjects were used in the training or model selection process. All data sets were preprocessed by performing centering and scaling. The parameters for this step were calculated for the training set and reused for the validation, test, and AIBL set.

### Feature selection

Initially, 103 volumes were extracted from the MRI scans. RF-importance was calculated for each MRI feature and the training set. The mean RF-importance of all features was used as a threshold. MRI features with an RF-importance smaller than this threshold were excluded from the data set. The feature selection was implemented using the Python library sci-kit [59] version 0.24.0 (https://scikit-learn.org/stable/, Accessed: 18 May 2021). The selected MRI features were expanded using three demographic features (age, gender, and count of ApoE *ε*4 alleles) and three cognitive test scores (MMSE, logical tests to evaluate the long-term memory (Logical memory, delayed – LDELTOTAL), and the short-term memory (Logical memory, immediate – LIMMTOTAL)).

### Data valuation based on Data Shapley or LOO

The idea of data valuation is to identify the subjects with the most informative data, restrict the training set to those subjects, and thus improve ML models. In this article, random subject exclusion, LOO [41] data valuation, and Data Shapley [15] are compared.

During random subject exclusion, subjects were randomly excluded from the data set without any previous valuation.

During the LOO and Data Shapley algorithms, ML models were trained to calculate the contribution of each subject. The entire training data set *D*={1,...,*n*} consists of *n* subjects. The performance of an ML model, trained with data set *D*, is denoted as *V*(*D*). In this work, *V*(*D*) is the classification accuracy for a predefined validation data set. The contribution of sample *i* on the overall model performance was called *Φ*_*i*_.

LOO data valuation calculates *Φ*_*i*_ as the accuracy difference of the ML models trained with and without a given subject *i*. This definition is formally described in Eq. 1.

1 $$ \Phi_{i}=V(D) -V\left(D\setminus\{i\}\right)  $$

Two ML models were trained to calculate the contribution of each subject. The first one was trained for the entire training set and the second one for the entire training set except for the subject of interest. However, this method lacked for principles of fairness. In this context, unfairness means that the sum of all individual contributions and the no-information rate is not equal to the overall model performance. Additionally, LOO is a greedy method that does not consider subject interactions.

Data Shapley values [15], which are based on Shapley values [36] (described in the “Shapley values” section), are one possibility to overcome this problem. Data Shapley values reach fairness by considering all subsets of subjects in the training data set and calculate a weighted sum of the individual contributions.

The computational effort for the exact calculation of Data Shapley values grows exponentially with the number of subjects *n* because a set of *n*-elements contains 2^*n*^−1 non-empty subsets. However, there are effective possibilities to estimate Data Shapley values. In this work, Truncated Monte Carlo (TMC) Shapley [15] was used.

The TMC algorithm starts with a random permutation of the training set. First, the performance of a random model is calculated. In this work, the accuracy for the predefined validation data set was used as the performance score. Afterwards, the randomly permuted subjects are successively added to the training data set, and ML models are trained. The contribution of the respectively added subject is calculated by subtracting the previously achieved validation performance from the validation performance of the new model. This procedure is repeated until new subjects reach only marginal improvements. The reason to use this truncation strategy is that subjects added at the beginning show higher contributions than subjects added later. Afterwards, the procedure is repeated with a new permutation. One contribution is thus calculated for each permutation and each subject.

The average contribution per subject estimates the Data Shapley values. The algorithm terminates if the calculated Shapley values meet a previously defined convergence criterion [15].

The pipeline of Ghorbani and Zou [15] (commit = 96e8ecb), available online (https://www.github.com/amiratag/DataShapley, Accessed: 18 May 2021) was used to implement the TMC Data Shapley algorithm. The experiments used logistic regression (LR) and RF models as base classifiers. Those models were implemented using the Python library sci-kit [59] version 0.24.0 (https://scikit-learn.org/stable/, Accessed: 18 May 2021). The hyperparameters followed the recommendations of the TMC Data Shapley implementation. In the experiments, four iterations of the TMC Data Shapley algorithm were performed with different seeds. The marginal contributions were averaged to generate a more robust model. The best-performing cutoff for the validation set was calculated using optimization. Subjects that achieved positive Data Shapley values improved the accuracy for the independent validation set, whereas subjects with negative Shapley values worsened the validation accuracy.

### Training of RF classifiers

RFs [42] were trained for the final classification between sMCI and pMCI subjects. RFs train multiple Decision Trees (DTs), each with a randomly selected subset of features and observations. The majority voting of those DTs predicts the final classification. The randomly selected features and observations make those models more robust and prevent overfitting. The RF algorithm was implemented using the Python sci-kit library [59] version 0.24.0 (https://scikit-learn.org/stable/, Accessed: 18 May 2021).

### Training of XGBoost classifiers

XGBoost [43] classifiers were trained in comparison to RF classifiers. XGBoost is a gradient-boosting model distributed as an open-source software library (https://xgboost.readthedocs.io/en/latest/, Accessed: 18 May 2021). The sequential combination of multiple weak classifiers into a strong joint classifier is the idea of boosting models. Gradient-boosting models fulfill this idea by training the initial classifier to learn the original dependent variable and the subsequent classifiers to learn the gradients of the previous classifier. The final model prediction is the sum of the weak classifiers. XGBoost is an implementation of gradient-boosting that promises scalability, parallelization, and distributed execution.

In the experiments, DTs were the base classifiers. The XGBoost classifier was implemented using the xgboost v1.2.0 Python library (https://xgboost.readthedocs.io/en/latest/python/python_intro.html, Accessed: 18 May 2021).

### Evaluation

The evaluation of the models was performed for the independent ADNI test set. None of those subjects was used during data valuation and model training. The performance of each model was evaluated using two metrics—namely accuracy (ACC) and F1-score (F1). The accuracy described in Eq. 2 measures the relative count of correctly classified subjects. The F1-score (F1) is described in Eq. 3. Table 3 shows the contingency table used to calculate the metrics.

2 $$ ACC=\frac{TP+TN}{TP+TN+FP+FN}  $$

**Table 3:** Contingency table for the classification between sMCI and pMCI subjects

Prediction	Diagnosis	
	pMCI	sMCI
pMCI	True positive (TP)	False positive (FP)
sMCI	False negative (FN)	True negative (TN)

3 $$ F1=\frac{TP}{TP+\frac{1}{2}(FP+FN)}  $$

### External validation

The external validation performed for the AIBL data set inspected the generalizability of the model. During data valuation and model training, the AIBL data set was not used.

### Interpretation model

For interpretation of black-box RF and XGBoost classifiers, Shapley values were used. In this context, the differences between the individual predictions and the average model prediction are explained by feature expressions.

The exact calculation of Shapley values for each subject and each feature requires multiple retraining of the ML black-box model. The computational effort exponentially increases with the number of features included in the models. For this reason, Kernel SHAP [44] was used to time-efficiently estimate Shapley values.

Kernel SHAP is based on Local Interpretable Model-agnostic Explanations (LIME) [60]. LIME are local surrogate models to interpret individual observations of black-box models. For each observation, LIME generate a new permutation of the training set. Then, the LIME algorithm fitted regression models to the weighted permutation data set. The weights depend on the distance from the observation at interest. Data points near this observation are weighted higher than data points far away.

Interpretable explanation models guaranteed interpretability. Eq. 4 shows the local optimization function of the LIME model. *L*(*f*,*g*,*π*_*x*_) is the loss function between the black-box model *f* and the local explanation model *g*. High complexity is prevented by using *Ω*(*g*). *π*_*x*_ defines the weighting of the observations.

4 $$ g^{*}(x)=\arg \min_{g\in G}L(f,g,\pi_{x})+\Omega(g)  $$

Kernel SHAP uses LIME to estimate Shapley values by fitting an additive linear model described in Eq. 5. In this equation, *x*^′^ is a simplified representation of the black-box model input features. For tabular data, the simplified features are binned binary feature representations. *M* is the total number of simplified features, and *Φ*_*i*_ are the Shapley values for each feature *i*.

5 $$ g(x')=\Phi_{0}+\sum_{i=1}^{M}\Phi_{i}\cdot x'_{i}  $$

The LIME parameters are described in Eqs. 6 and 7 and further derived in Lundberg and Lee [44]. Additionally, *Ω*(*g*) is set to zero. *h*_*x*_(*x*^′^)=*x* maps the simplified input features *x*^′^ to the original feature space.

6 $$ \pi_{x}(x')=\frac{M-1}{ {M \choose {|x'|}}\cdot|x'|\cdot(M-|x'|)}  $$

7 $$ L(f,g,\pi_{x'})=\sum_{x'\in X}\Big(f\big(h_{x}(x')\big)-g(x')\Big)^{2}\cdot\pi_{x'}(x')  $$

Kernel SHAP is part of the SHAP framework [44]. In this framework, three properties—namely local accuracy, missingness, and consistency—are described. SHAP Values fulfilled all three criteria.

The Python library SHAP, version 0.38.1 (https://github.com/slundberg/shap, Accessed: 18 May 2021), was used to implement the Kernel SHAP explanation method.

## Results

This section describes the results of the experiments based on the ML workflow. Data Shapley values and LOO values were calculated using RF and LR base classifiers. However, RF and XGBoost classifiers were used to train the final ML models. The base classifier used to calculate Data Shapley values are exclusively used in front of the “Data Shapley values” or “Data Shapley method” terms to avoid confusion. Thus, for example, Data Shapley values calculated with an LR base classifier are denoted as LR Data Shapley values.

### Feature selection

The RF-importance filter strategy selected 33 of the initially 103 MRI features. The selected features and the RF-importances are summarized in Table 4. The most important features were volumes of the left hippocampus, the left entorhinal cortex, and the right amygdala. Most of those features were previously associated with AD progression [61–64].

**Table 4:** RF feature importance calculated for the selected MRI volumetric features using an RF-importance filter strategy

Anatomical brain structure	Feature name	RF-importance
Left hippocampus	Left-Hippocampus	0.035
Left entorhinal cortex	lh_entorhinal_volume	0.033
Right amygdala	Right-Amygdala	0.032
Left middle temporal gyrus	lh_middletemporal_volume	0.029
Left amygdala	Left-Amygdala	0.028
Right hippocampus	Right-Hippocampus	0.026
Right entorhinal cortex	rh_entorhinal_volume	0.021
Left fusiform gyrus	lh_fusiform_volume	0.020
Right banks of superior temporal sulcus	rh_bankssts_volume	0.019
Left supramarginal gyrus	lh_supramarginal_volume	0.019
Right middle temporal gyrus	rh_middletemporal_volume	0.017
Right fusiform gyrus	rh_fusiform_volume	0.017
Left superior parietal lobule	lh_superiorparietal_volume	0.015
Left inferior parietal lobule	lh_inferiorparietal_volume	0.014
Left banks of superior temporal sulcus	lh_bankssts_volume	0.014
Right cortex	rhCortexVol	0.013
Left inferior temporal gyrus	lh_inferiortemporal_volume	0.013
Right nucleus accumbens area	Right-Accumbens-area	0.012
Left insular cortex	lh_insula_volume	0.012
Left cuneus	lh_cuneus_volume	0.012
Right inferior parietal lobule	rh_inferiorparietal_volume	0.011
Left transverse temporal gyrus	lh_transversetemporal_volume	0.010
Left pars opercularis	lh_parsopercularis_volume	0.010
Left pericalcarine cortex	lh_pericalcarine_volume	0.010
Left superior frontal gyrus	lh_superiorfrontal_volume	0.010
Left posterior cingulate cortex	lh_posteriorcingulate_volume	0.010
Left isthmus of cingulate gyrus	lh_isthmuscingulate_volume	0.010
Right inferior lateral ventricle	Right-Inf-Lat-Vent	0.010
Right isthmus of cingulate gyrus	rh_isthmuscingulate_volume	0.010
Right thalamus proper	Right-Thalamus-Proper	0.010
Left globus pallidus	Left-Pallidum	0.010
Right superior frontal gyrus	rh_superiorfrontal_volume	0.010
Right insular cortex	rh_insula_volume	0.010

### Data valuation based on Data Shapley or LOO

This section investigates data valuation results achieved using Data Shapley values. Subjects with high Data Shapley values were identified as having the most informative data. The data set was split between diagnostic groups after the calculation of LR and RF Data Shapley values. An RF model to predict Data Shapley values was trained for each group. For each Data Shapley base classifier and each diagnostic group, one SHAP summary plot was created. Those plots investigated the associations between Data Shapley values and feature values. The features were sorted based on the RF feature importance. For the sMCI group, it was expected that subjects with large brain volumes [61, 65–67], no ApoE *ε*4 alleles [68–70] and good performances in cognitive tests [71] were more representative and would reach higher Data Shapley values. Consistently, it was expected that MCI subjects with small brain volumes, one or two ApoE *ε*4 alleles and bad performances in cognitive tests more likely convert to AD and would thus reach higher Data Shapley values.

**Fig. 4:**
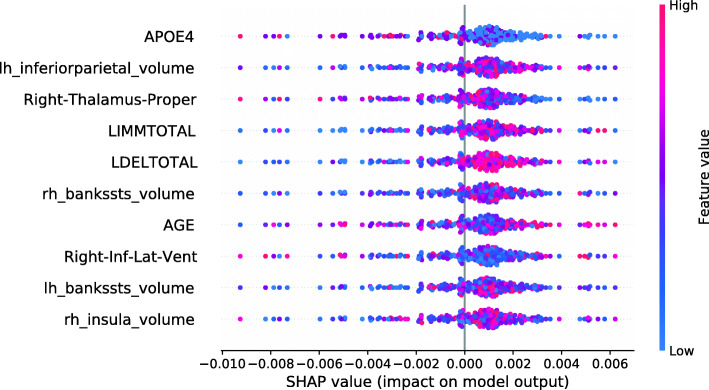
SHAP summary plot, visualizing the LR Data Shapley values of the sMCI diagnostic group dependent on the feature values. Each point visualizes a Data Shapley value for one subject and one feature. The color of the points depends on the feature values, and the horizontal axis shows the calculated Shapley values. The vertical axis represents both the features, ordered by the mean absolute Shapley values and their distribution

Figure 4 shows the SHAP summary plot for the LR Data Shapley values and the sMCI diagnostic group. The number of ApoE *ε*4 alleles was the most important feature to predict the LR Data Shapley values in the sMCI group. The absence of ApoE *ε*4 alleles (colored in blue) was associated with high LR Data Shapley values. Smaller LR Data Shapley values were reached for subjects with one (colored in purple) or two (colored in red) ApoE *ε*4 alleles. The second most important feature was the volume of the left inferior parietal lobule. For this feature, small volumes (colored in blue) were mainly associated with small LR Data Shapley values. However, some subjects with small volumes of the left inferior parietal lobule reached high LR Data Shapley values. This indicated that the LR Data Shapley values were associated with complex patterns and depended on many features. The volume of the right thalamus proper was the third most important feature to predict the LR Data Shapley values in the sMCI group. Small brain volumes were associated with high LR Data Shapley values. The LDELTOTAL and LIMMTOTAL cognitive test scores showed those good test performances (colored in red) were associated with high LR Data Shapley values, and poor test performances were associated with small LR Data Shapley values. The sMCI subject that reached the smallest LR Data Shapley value had two ApoE *ε*4 alleles (colored in red), bad performances in the cognitive tests, a rather young age of 65.7 years, and a complex pattern of the MRI features. The combination of poor test performances, two ApoE *ε*4 alleles, and young age might cause the small LR Data Shapley value of this subject.

**Fig. 5:**
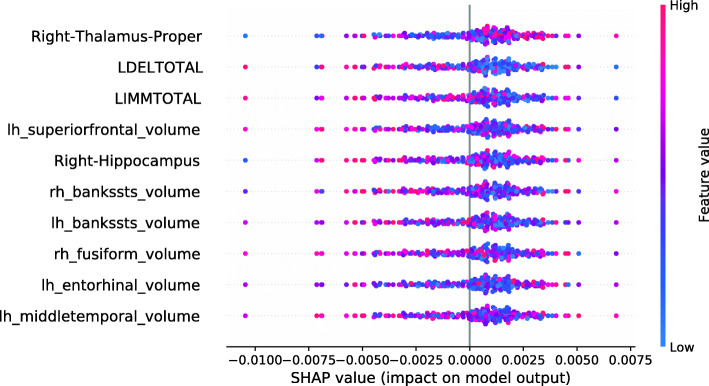
SHAP summary plot, visualizing the LR Data Shapley values of the pMCI diagnostic group dependent on the feature values. Each point visualizes a Data Shapley value for one subject and one feature. The color of the points depends on the feature values, and the horizontal axis shows the calculated Shapley values. The vertical axis represents both the features, ordered by the mean absolute Shapley values and their distribution

The SHAP summary plot shown in Fig. 5 visualizes the association of the LR Data Shapley values and the feature values in the pMCI group. The volume of the right thalamus proper was the most important feature to predict the LR Data Shapley values in the pMCI group. It was surprisingly noted that high brain volumes were associated with high LR Data Shapley values. The LDELTOTAL cognitive test score was the second most important feature in this plot. Poor test performances (colored in blue) were associated with positive LR Data Shapley values. A similar observation can be seen for the LIMMTOTAL cognitive test score, which was the third most important feature in this plot. The pMCI subject with the smallest LR Data Shapley value had good cognitive test scores, and rather high volumetric feature values, except for the right thalamus proper, and the right hippocampus. The small LR Data Shapley value might be caused by good test performances and high brain volumes, which were less representative for pMCI subjects.

**Fig. 6:**
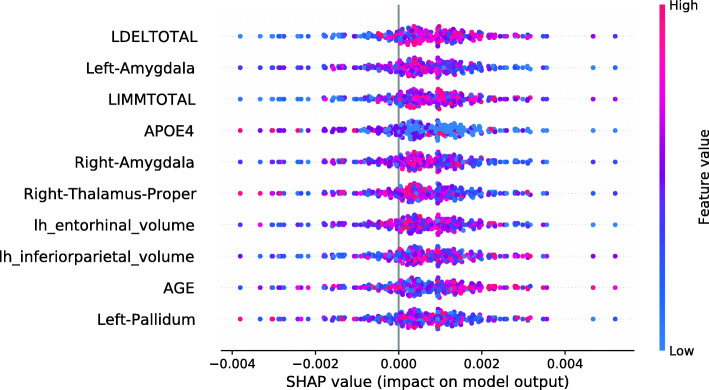
SHAP summary plot, visualizing the RF Data Shapley values of the sMCI diagnostic group dependent on the feature values. Each point visualizes a Data Shapley value for one subject and one feature. The color of the points depends on the feature values, and the horizontal axis shows the calculated Shapley values. The vertical axis represents both the features, ordered by the mean absolute Shapley values and their distribution

The SHAP summary plot in Fig. 6 shows the associations between the feature values and the RF Data Shapley values in the sMCI diagnostic group. The most important feature in this plot was the LDELTOTAL cognitive test score. High cognitive test scores were associated with high RF Data Shapley values, and small test scores were associated with small RF Data Shapley values. The same applied to the LIMMTOTAL cognitive test score, which was the third most important feature. The second most important feature was the volume of the left amygdala. Small volumes of the left amygdala were associated with both large and small RF Data Shapley values. High volumes of the left amygdala were associated with medium RF Data Shapley values. The sMCI subject with the smallest RF Data Shapley value was rather young (65.7 years) and had bad cognitive test performances, two ApoE *ε*4 alleles and medium to high brain volumes. The small RF Data Shapley value might result from the two ApoE *ε*4 alleles and the bad cognitive test results. This subject also achieved the smallest LR Data Shapley value in the sMCI group.

**Fig. 7:**
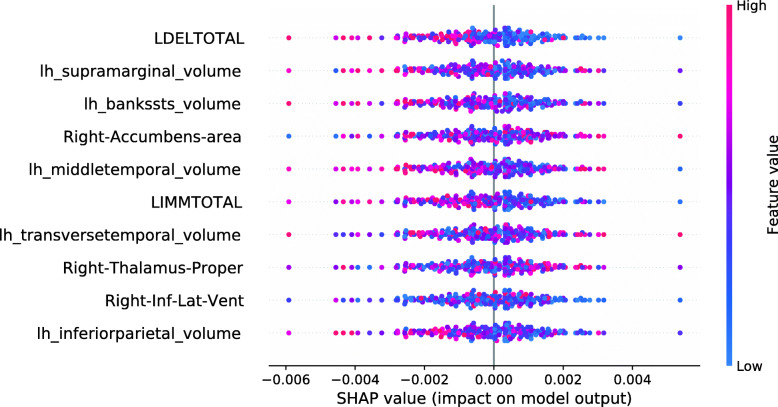
SHAP summary plot, visualizing the RF Data Shapley values of the pMCI diagnostic group dependent on the feature values. Each point visualizes a Data Shapley value for one subject and one feature. The color of the points depends on the feature values, and the horizontal axis shows the calculated Shapley values. The vertical axis represents both the features, ordered by the mean absolute Shapley values and their distribution

The SHAP summary plot visualized in Fig. 7 shows the associations between feature values and RF Data Shapley values in the pMCI group. For the pMCI group, the LDELTOTAL cognitive test score was the most important feature. Poor test performances were associated with high RF Data Shapley values, and subjects with high LDELTOTAL test scores reached small RF Data Shapley values. The second most important feature was the volume of the left supramarginal gyrus. Subjects with small brain volumes mainly achieved positive Data Shapley values. The pMCI subject with the smallest RF Data Shapley value was a subject with high performance in the cognitive tests and high brain volumes except for the right nucleus accumbens area. The small RF Data Shapley value might be associated with good cognitive test performances and high brain volumes. This pattern is less representative for pMCI subjects.

### Training of RF classifiers

**Fig. 8:**
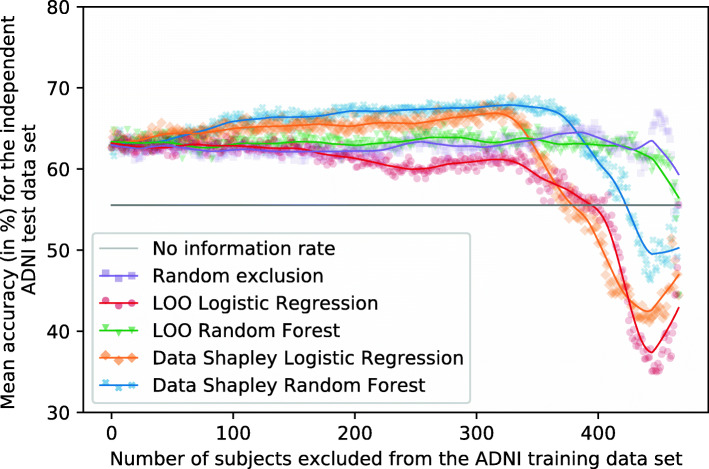
Plot showing the mean RF accuracies for the independent ADNI test set (no information rate 55.56%). Different methods were used to identify and focus on the training subjects (*n* = 467) with the most informative data. Ten repetitions with different seeds were performed for every exclusion data set

**Fig. 9:**
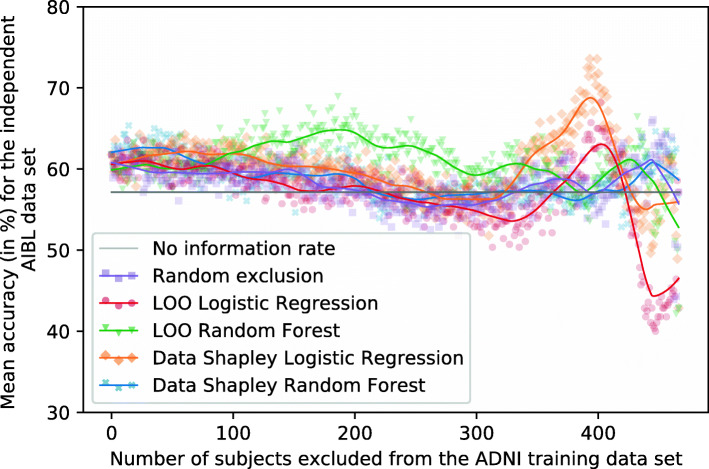
Plot showing the mean RF accuracies for the external AIBL test set (no information rate 57.14%). Different methods were used to identify and focus on the training subjects (*n* = 467) with the most informative data. Ten repetitions with different seeds were performed for every exclusion data set

This section compares the results of RF models that exclude subjects based on different data valuation techniques. Therefore, subjects with the smallest contributions are successively excluded from the training set. Ten RF models were trained with the associated training set to reach more robust results. Each of those ten models was trained with a different seed. The performances are mean accuracies and F1-scores for the independent test set. Figures 8 and 9 visualize the mean RF accuracies dependent on the number of subjects excluded from the training set and the data valuation strategy. Tables 5 and 7 summarize those results for predefined numbers of subjects excluded from the training set. Additionally, Tables 6 and 8 summarize the mean accuracies and F1-scores achieved for the test set by excluding all subjects with negative Data Shapley values from the data set, for the maximum exclusion cutoff determined for the validation set, and the maximum exclusion cutoff determined for the test set. However, the maximum exclusion cutoff for the test set was not validated for an independent test set and is thus an optimistic estimation. The models which excluded all subjects with Data Shapley values smaller than zero were called zero-cutoff models. The idea of the zero-cutoff is that subjects with negative Data Shapley values decreased the classification results for the validation set. 164 subjects reached RF Data Shapley values smaller than zero, and 152 subjects reached LR Data Shapley values smaller than zero.

**Table 5:** RF accuracies (mean ± standard deviation in %) for the independent ADNI test set (no information rate 55.56%)

Exclusion method	Number of training subjects excluded					
(base model)	0	50	100	150	200	250
Random (-)	**62.64 ±0.87**	62.29 ±0.93	61.46 ±1.87	61.11 ±1.49	62.36 ±1.61	63.47 ±2.31
LOO (LR)	**62.64 ±0.87**	61.53 ±1.87	63.47 ±1.08	62.92 ±1.73	61.46 ±1.36	59.72 ±1.58
LOO (RF)	**62.64 ±0.87**	63.68 ±1.99	62.36 ±2.19	63.68 ±1.86	62.71 ±1.08	63.96 ±1.95
Data Shapley (LR)	**62.64 ±0.87**	**64.72 ±0.87**	64.93 ±0.78	65.21 ±1.26	65.62 ±1.29	65.00 ±2.24
Data Shapley (RF)	**62.64 ±0.87**	63.68 ±1.76	**66.25 ±0.89**	**66.46 ±1.28**	**66.67 ±1.16**	**67.29 ±1.18**

Different methods were used to identify and focus on the training subjects with the most informative data. Ten repetitions with different seeds were performed for every exclusion data set. The best results are highlighted in bold

**Table 6:** Mean RF accuracies and F1-scores (in %) for the independent ADNI test set (no information rate 55.56%)

Exclusion method	Zero-cutoff			Max valid			Max test		
	*n*	ACC (in %)	F1 (in %)	*n*	ACC (in %)	F1 (in %)	*n*	ACC (in %)	F1 (in %)
Random (-)	–	–	–	41	63.47	66.49	449	67.01	73.77
LOO (LR)	84	63.06	65.75	102	64.31	66.70	5	64.44	68.23
LOO (RF)	49	63.47	66.99	23	63.33	67.09	273	65.42	68.50
Data Shapley (LR)	152	65.76	68.78	340	65.49	67.18	329	**68.89**	70.33
Data Shapley (RF)	164	**66.88**	**72.21**	134	**66.25**	**70.96**	321	68.82	**74.53**

Different methods were used to identify and focus on the training subjects with the most informative data. The zero-cutoff method excluded all training subjects with Data Shapley values smaller than zero. Max valid was the threshold achieved by maximizing the results for the independent validation set. Max test was the optimistic threshold which achieved the best results for the test set. Ten repetitions with different seeds were performed for every exclusion data set. The best results are highlighted in bold

**Table 7:** RF accuracies (mean ± standard deviation in %) for the external AIBL data set (no information rate 57.14%)

Exclusion method	Number of training subjects excluded					
(base model)	0	50	100	150	200	250
Random (-)	**61.79 ±3.93**	61.43 ±5.00	59.29 ±2.37	61.79 ±2.79	58.21 ±2.29	53.21 ±3.37
LOO (LR)	**61.79 ±3.93**	57.86 ±3.50	58.93 ±4.30	55.71 ±3.64	56.79 ±5.40	59.29 ±3.98
LOO (RF)	**61.79 ±3.93**	57.50 ±6.07	**63.57 ±3.11**	**62.14 ±5.80**	**62.86 ±5.80**	**64.29 ±2.77**
Data Shapley (LR)	**61.79 ±3.93**	**63.57 ±4.74**	60.71 ±4.23	60.36 ±1.92	58.57 ±5.10	57.86 ±3.85
Data Shapley (RF)	**61.79 ±3.93**	63.21 ±3.21	61.43 ±2.67	58.93 ±2.88	57.50 ±4.06	55.36 ±1.79

Different methods were used to identify and focus on the training subjects with the most informative data. Ten repetitions with different seeds were performed for every exclusion data set. The best results are highlighted in bold

**Table 8:** Mean RF accuracies and F1-scores (in %) for the AIBL data set (no information rate 57.14%)

Exclusion method	Zero-cutoff			Max valid			Max test		
	*n*	ACC (in %)	F1 (in %)	*n*	ACC (in %)	F1 (in %)	*n*	ACC (in %)	F1 (in %)
Random (-)	–	–	–	41	58.57	66.42	445	66.07	75.81
LOO (LR)	84	**62.50**	68.81	102	56.79	64.44	402	68.21	76.33
LOO (RF)	49	57.86	65.40	23	**61.79**	69.45	186	68.93	**76.63**
Data Shapley (LR)	152	61.79	**69.52**	340	61.07	65.79	399	**73.57**	75.74
Data Shapley (RF)	164	57.86	68.94	134	60.36	**70.05**	14	65.36	72.30

Different methods were used to identify and focus on the training subjects with the most informative data. The zero-cutoff method excluded all training subjects with Data Shapley values smaller than zero. Max valid was the threshold achieved by maximizing the results for the independent validation set. Max test was the optimistic threshold which achieved the best results for the test set. Ten repetitions with different seeds were performed for every exclusion data set. The best results are highlighted in bold

#### Evaluation

Figure 8 shows the mean RF accuracies for the ADNI test set dependently on the number of subjects excluded from the training set and the data valuation strategies. Tables 5 and 6 summarize those results. The no information rate of the ADNI test set was 55.56%, and the base models trained on the entire training set reached a mean accuracy of 62.64%.

If those subjects with the smallest RF and LR Data Shapley values were excluded from the training set, improved classification results can be recognized. The increase of the RF Data Shapley method was slightly higher than the LR Data Shapley results. However, the overall best results on the test set were reached by excluding those 329 subjects with the smallest LR Data Shapley values. Those models reached a mean accuracy of 68.89% and an F1-score of 70.33%. The LR Data Shapley method found an optimum for the validation set by excluding 340 training subjects. The associated model reached a mean accuracy of 65.49% and thus outperformed the base model by 4.55% (2.85 percentage points). The RF model that excluded subjects with LR Data Shapley values smaller than zero reached a mean accuracy of 65.76% and was thus 4.98% (3.12 percentage points) better than the base model. The LR Data Shapley exclusion strategy achieved results smaller than the no information rate after approximately 375 training subjects were excluded.

The RF Data Shapley method outperformed all the other methods between cutoff values of approximately 75 and 375. The best result for the RF Data Shapley method was 68.82%, reached by excluding 321 training subjects. This model achieved a mean F1-score of 74.53%. The optimization process, which was executed for the validation set, excluded 134 training subjects and reached a mean accuracy of 66.25%. The RF Data Shapley methods performed worse than the no information rate of the ADNI data set after approximately 425 subjects were excluded. The model trained on those 164 subjects with positive RF Data Shapley values reached a mean accuracy of 66.88% and was thus 6.77% (4.24 percentage points) better than the base model. The best mean accuracy of the random exclusion method was 67.01%, which was 2.73% (1.88 percentage points) worse than the best LR Data Shapley results. This result was achieved by randomly excluding 449 and thus 96.15% of the training subjects. A disadvantage of this model was that it included only a few data samples. Thus, the risk of a selection bias was large, which increases the risk of less robust performances for other cohorts. Overall, for the Data Shapley method, improved performances for the independent ADNI test set were observed.

#### External validation

It can be seen in Fig. 9 that the scattering of the classification results for the external AIBL data set is higher than for the independent ADNI test set. Tables 7 and 8 summarize the results visualized in this figure. The no information rate of the AIBL data set was 57.14%, and the base model reached a mean accuracy of 61.79%. The RF Data Shapley method showed a slight increase of accuracies by excluding between 0 and 75 training subjects. The maximum mean accuracy was achieved by excluding those 14 subjects with the smallest RF Data Shapley values. The mean accuracy of those models was 65.36%, and the mean F1-score was 72.30%. After this peak, the accuracies of the RF Data Shapley method decreased except for a small peak by excluding almost all training subjects. The validation cutoff value was 134 for this method, and the associated models reached a mean accuracy of 60.36% for this threshold. This accuracy was 2.30% (1.42 percentage points) smaller than the base model performance.

The LR Data Shapley method had a course that was similar to the RF Data Shapley exclusion method, except for a high peak by excluding between 350 and 425 subjects from the training set. The best model, which excluded 399 training subjects, reached an accuracy of 73.57% and an F1-score of 75.74%. The cutoff value which was calculated for the validation set was 340. The associated models reached a mean accuracy of 61.07%. This result was smaller than the base model performance.

The random exclusion method and the LR LOO method had a slightly decreasing course for the AIBL data set. The RF LOO method outperformed all the other methods by excluding between 100 and 325 subjects. The best accuracy of 68.93% was achieved by excluding 186 subjects from the training set.

#### Interpretation model

**Fig. 10:**
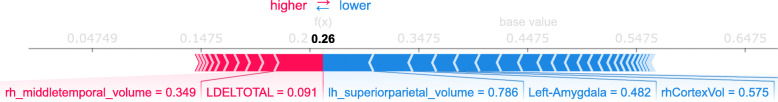
SHAP force plot for an ADNI sMCI subject (PTID = 123_S_4904) from the training set to explain the prediction of the RF base model. SHAP force plots show that the individual prediction (f(x) = 0.26) consists of the sum of all feature Shapley values and the average model prediction (base value = 0.4475). The associated Shapley value of a feature is visualized using the length of an arrow. Feature expressions with large Shapley values have strong effects on the individual prediction and are shown in the middle of SHAP force plots. Pathogenic feature expressions with Shapley values higher than zero are shown as red and protective expressions as blue arrows. The MRI volumetric features and the cognitive test scores were min-max-normalized

SHAP force plot explain individual model predictions, which are important in clinical practice. Figure 10 shows a SHAP force plot for the RF base model and the sMCI training subject with the PTID 123_S_4904. This sMCI subject reached the smallest LR and RF Data Shapley values and was identified as a subject with less informative data. The average model prediction for this RF model was 0.4475, and the model prediction probability for the visualized subject was 0.26. The SHAP force plot explains the difference between those two values using the model features. The lengths of the arrow parts in this plot demonstrate the Kernel SHAP values of those features. Feature expressions with positive Kernel SHAP values and thus a pathogenic effect on the overall prediction are colored in red and feature expressions with a negative Kernel SHAP value had a protective effect and are colored in blue. For the subject visualized in Fig. 10, the most important feature was the volume of the left superior parietal lobule. The subject had a min-max-scaled volume of 0.786, which is a rather high volume. The model learned that this feature expression had a protective effect on this subject. Therefore, the risk of this subject converting to AD was decreased by the small superior parietal lobule volume. The reduction of GM was previously associates with AD progression in the superior parietal lobule [72]. The LDELTOTAL cognitive test score was the most important feature with a pathogenic effect on the prediction probability for this subject. The normalized volume of this feature was 0.091 and thus a poor test performance.

**Fig. 11:**
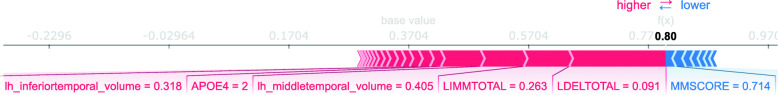
SHAP force plot for an ADNI sMCI subject (PTID = 123_S_4904) from the training set to explain the prediction of the RF model, trained by excluding those 134 training subjects with the smallest LR Data Shapley values. SHAP force plots show that the individual prediction (f(x) = 0.80) consists of the sum of all feature Shapley values and the average model prediction (base value = 0.3704). The length of an arrow visualized the associated Shapley value of a feature. Feature expressions with large Shapley values have strong effects on the individual prediction and are shown in the middle of SHAP force plots. Pathogenic feature expressions have Shapley values higher than zero and are visualized as red arrows. Protective expressions are visualized as blue arrows. The MRI volumetric features and the cognitive test scores were min-max-normalized

Figure 11 shows a SHAP force plot for the same subject, but an RF model trained on all training subjects except for those 134 subjects with the smallest LR Data Shapley values. 134 was the cutoff value that reached the best mean accuracy for the validation set. This model misclassified the subject as a pMCI subject with a probability of 0.80. The prediction in this classification model is based on cognitive test scores. The most important feature expression for this prediction was the bad performance in the LDELTOTAL cognitive test. The min-max-scaled LDELTOTAL score of this subject was 0.091 (unscaled: 1). The model learned that the poor test performance increased the subject’s risk of converting to AD. The most important feature in this model with a protective effect was the MMSE cognitive test score, which had a high min-max-normalized value of 0.714 (unscaled: 28). It can be inferred that this subject reached a small Data Shapley value because of the bad performance in LDELTOTAL and LIMMTOTAL cognitive tests and two ApoE *ε*4 alleles and young age (65.7 years), which is not visualized in this plot. This combination might suggest that this subject will prospectively convert to AD. It can be seen that the model which excluded 134 subjects from the training set focussed more on cognitive test scores for this subject and which might cause a more robust model.

SHAP summary plots summarize the explanations of the training, test, and validation subjects. Overall, due to the atrophy pattern of AD, it was expected that large brain volumes had a protective, and small brain volumes had a pathogenic effect on the disease progression [61, 65–67]. Additionally, an enlargement of the ventricles was expected [73, 74] during the disease progression. As ApoE *ε*4 is a risk factor of AD, it was expected that the presence of ApoE *ε*4 alleles increases the risk to develop AD [68–70].

**Fig. 12:**
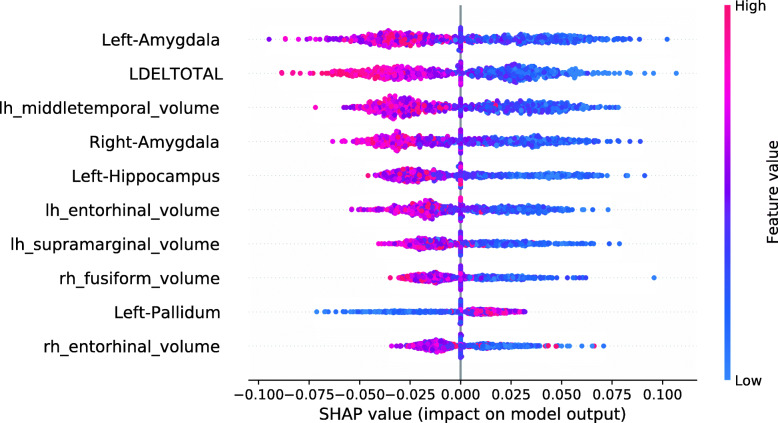
SHAP summary plot for the RF model. No training subjects were excluded. SHAP summary plots aggregate the explanation of individual predictions of the entire training and test set. Each point visualizes a Shapley value for a subject and a feature. The color of the points depends on the feature values, and the horizontal axis shows the calculated Shapley values. The vertical axis represents both the features, ordered by the mean absolute Shapley values and their distribution. The positive class is pMCI

The SHAP summary plot in Fig. 12 shows that the most important model feature was the volume of the left amygdala. The model found that small brain volumes mainly had a pathogenic effect on the subject’s prediction. Consistently, the model learned that high brain volumes decreased the risk of a subject converting to AD. The second most important feature in this model was the LDELTOTAL cognitive test score. The model learned that high test performances (colored in red) had a protective effect on developing AD. Poor test performances (colored in blue) instead had a pathogenic effect on the development of AD.

The model shows the learned associations for the ten most important features. All of those features except for the volume of the left pallidum showed biologically plausible associations, as small brain volumes and bad performances in cognitive tests were associated with disease progression.

**Fig. 13:**
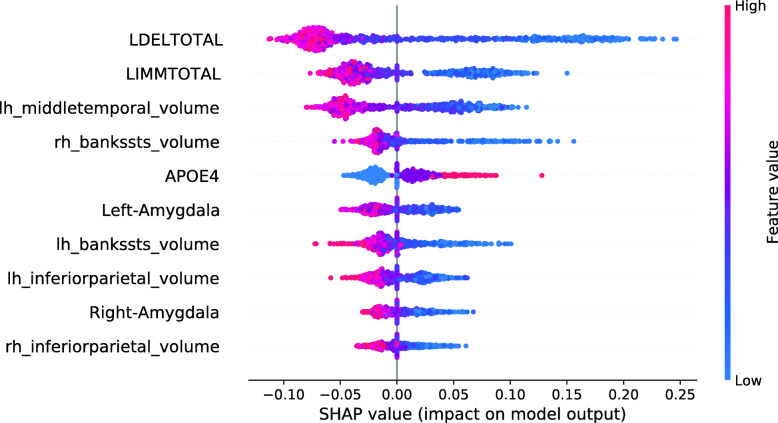
SHAP summary plot for the RF model. 134 training subjects with the smallest RF Data Shapley values were excluded. SHAP summary plots aggregate the explanation of individual predictions of the entire training and test set. Each point visualizes a Shapley value for a subject and a feature. The color of the points depends on the feature values, and the horizontal axis shows the calculated Shapley values. The vertical axis represents both the features, ordered by the mean absolute Shapley values and their distribution. The positive class is pMCI

Figure 13 shows the SHAP summary plot for an RF model trained on the entire training set, except for those 134 subjects with the smallest RF Data Shapley values. 134 was the cutoff value optimized for the validation set and the RF Data Shapley method. The most important feature in this model was the LDELTOTAL cognitive test score. The model learned that high test scores had a protective effect on the subjects prediction and small test scores had a pathogenic effect. The same applies to the LIMMTOTAL cognitive test score, which was the second most important feature in this model. The volume of the left middle temporal gyrus was the third most important feature. The model learned that small volumes increased the subject’s risk of converting to AD, whereas high volumes had a protective effect. In comparison to the model trained on the entire training set, the number of ApoE *ε*4 alleles was more important in this model. The model learned that the presence of ApoE *ε*4 alleles was associated with AD progression. None of the associations visualized in Fig. 13 showed a biologically implausible behavior. Overall, cognitive test scores were more relevant in the model trained for the reduced training set.

### Training of XGBoost classifiers

**Fig. 14:**
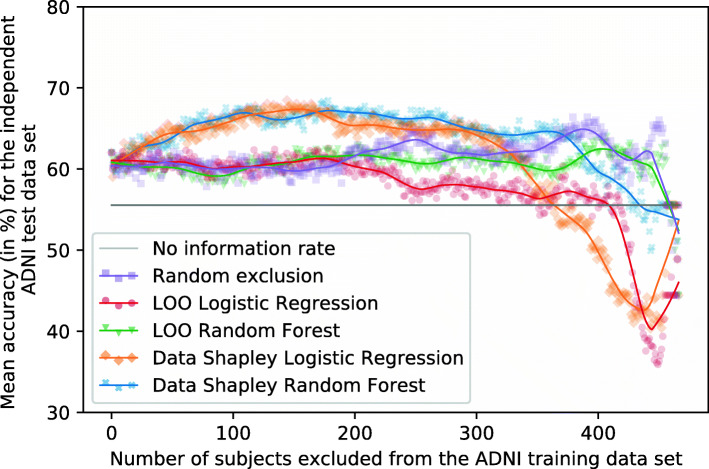
Plot showing the mean XGBoost accuracies for the independent ADNI test set (no information rate 55.56%). Different methods were used to identify and focus on the training subjects (*n* = 467) with the most informative data. Ten repetitions with different seeds were performed for every exclusion data set

**Fig. 15:**
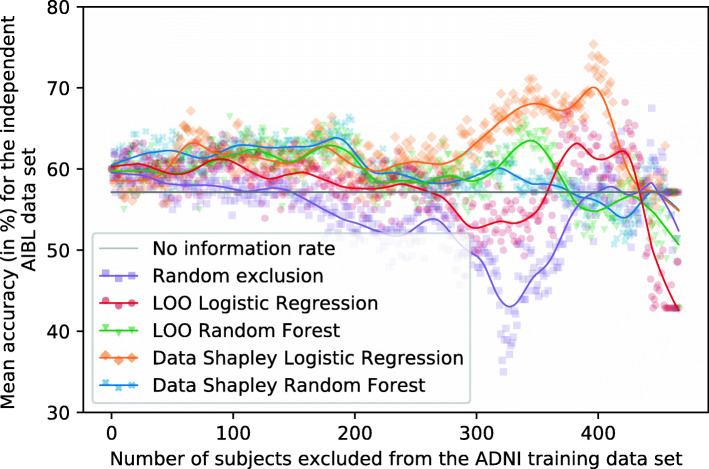
Plot showing the mean XGBoost accuracies for the external AIBL test set (no information rate 57.14%). Different methods were used to identify and focus on the training subjects (*n* = 467) with the most informative data. Ten repetitions with different seeds were performed for every exclusion data set

This section describes the results for the XGBoost models achieved by excluding subjects based on different data valuation techniques. The experiments correspond to those executed for the RF classifier. Figures 14 and 15 visualize the mean XGBoost accuracies dependent on the number of training subjects excluded and the data valuation strategy. Tables 9 and 11 summarize the results for predefined cutoff values. Tables 10 and 12 summarize the mean accuracies and F1-scores for highlighting cutoffs of the test and validation data set. However, the maximum exclusion cutoff for the test set was not validated for an independent test set and is thus an optimistic estimation.

**Table 9:** Mean XGBoost accuracies (mean ± standard deviation in %) for the independent ADNI test set (no information rate 55.56%)

Exclusion method	Number of training subjects excluded					
(base model)	0	50	100	150	200	250
Random (-)	**62.01 ±1.59**	60.42 ±1.28	59.51 ±2.54	59.79 ±1.37	62.57 ±1.50	64.58 ±1.42
LOO (LR)	**62.01 ±1.59**	60.14 ±1.73	59.72 ±1.91	61.46 ±1.68	59.03 ±1.89	56.94 ±1.58
LOO (RF)	**62.01 ±1.59**	61.04 ±2.35	58.54 ±1.59	61.11 ±1.64	61.74 ±2.25	59.72 ±2.04
Data Shapley (LR)	**62.01 ±1.59**	**64.72 ±1.58**	**66.88 ±1.39**	**67.22 ±1.48**	64.65 ±1.14	64.58 ±1.20
Data Shapley (RF)	**62.01 ±1.59**	63.61 ±1.79	66.18 ±1.55	66.81 ±1.83	**67.15 ±1.20**	**66.46 ±1.12**

Different methods were used to identify and focus on the training subjects with the most informative data. Ten repetitions with different seeds were performed for every exclusion data set. The best results are highlighted in bold

**Table 10:** Mean XGBoost accuracies and F1-scores (in %) for the independent ADNI test set (no information rate 55.56%)

Exclusion method	Zero-cutoff			Max valid			Max test		
	*n*	ACC (in %)	F1 (in %)	*n*	ACC (in %)	F1 (in %)	*n*	ACC (in %)	F1 (in %)
Random (-)	–	–	–	430	62.78	64.45	391	66.39	68.40
LOO (LR)	84	60.56	63.50	69	60.62	65.05	178	63.57	65.28
LOO (RF)	49	61.25	65.84	11	59.38	63.96	407	64.03	68.21
Data Shapley (LR)	152	**68.06**	71.02	248	65.07	67.89	144	**68.47**	71.22
Data Shapley (RF)	164	66.88	**72.12**	133	**65.90**	**70.62**	178	**68.47**	**73.48**

Different methods were used to identify and focus on the training subjects with the most informative data. The zero-cutoff method excluded all training subjects with Data Shapley values smaller than zero. Max valid was the threshold achieved by maximizing the results for the independent validation set. Max test was the optimistic threshold which achieved the best results for the test set. Ten repetitions with different seeds were performed for every exclusion data set. The best results are highlighted in bold

**Table 11:** XGBoost accuracies (mean ± standard deviation in %) for the external AIBL data set

Exclusion method	Number of training subjects excluded					
(base model)	0	50	100	150	200	250
Random (-)	**60.00 ±5.00**	55.00 ±3.98	57.50 ±4.06	55.71 ±2.37	52.14 ±5.35	51.07 ±4.80
LOO (LR)	**60.00 ±5.00**	58.93 ±3.29	61.79 ±4.53	**63.21 ±3.93**	58.21 ±3.93	59.64 ±4.80
LOO (RF)	**60.00 ±5.00**	59.64 ±5.99	61.43 ±5.71	61.79 ±2.79	61.07 ±4.64	56.07 ±4.53
Data Shapley (LR)	**60.00 ±5.00**	61.07 ±3.73	61.43 ±3.85	62.14 ±3.98	**63.21 ±5.31**	**61.07 ±1.92**
Data Shapley (RF)	**60.00 ±5.00**	**64.64 ±5.64**	**62.86 ±2.86**	62.86 ±1.75	61.79 ±2.29	58.57 ±2.37

Different methods were used to identify and focus on the training subjects with the most informative data. Ten repetitions with different seeds were performed for every exclusion data set. The best results are highlighted in bold

**Table 12:** Mean XGBoost accuracies and F1-scores (in %) for the AIBL data set (no information rate 57.14%)

Exclusion method	Zero-cutoff			Max valid			Max test		
	*n*	ACC (in %)	F1 (in %)	*n*	ACC (in %)	F1 (in %)	n	ACC (in %)	F1 (in %)
Random (-)	–	–	–	430	55.00	57.97	444	67.50	73.44
LOO (LR)	84	59.64	66.21	69	**63.21**	70.00	422	68.21	73.68
LOO (RF)	49	57.14	64.69	11	61.07	68.43	97	66.43	73.53
Data Shapley (LR)	152	**63.21**	69.97	248	58.57	66.66	396	**75.36**	**77.53**
Data Shapley (RF)	164	62.14	**71.65**	133	61.79	**70.84**	195	66.43	75.34

Different methods were used to identify and focus on the training subjects with the most informative data. The zero-cutoff method excluded all training subjects with Data Shapley values smaller than zero. Max valid was the threshold achieved by maximizing the results for the independent validation set. Max test was the optimistic threshold which achieved the best results for the test set. Ten repetitions with different seeds were performed for every exclusion data set. The best results are highlighted in bold

#### Evaluation

Figure 14, Tables 9 and 10 summarize the mean XGBoost accuracies dependent on the data valuation strategies and the number of subjects excluded from the training set for the independent ADNI test set. The no information rate for the ADNI test set was 55.56%. The mean accuracy of the base model was 62.01% which was slightly better than the results achieved for the RF models presented in Fig. 8. For both base models, the mean accuracies increased if training subjects with small Data Shapley values were excluded. The LR Data Shapley method showed increased accuracies until the maximum of 68.47% was reached by excluding 144 subjects. After this maximum, the classification accuracies decreased. After approximately 325 training subjects were excluded, the model achieved results worse than the random exclusion method and worse than the no information rate after approximately 375 subjects were excluded. The validation cutoff value was 248 for this method, and the associated model reached an accuracy of 65.07%.

The best mean accuracy for the RF Data Shapley method was 68.47%, reached by excluding 178 training subjects. This model reached an F1-score of 73.48%. The validation cutoff was 133, and the models trained with this cutoff value reached a mean accuracy of 65.90%. This value was 3.75% (2.57 percentage points) worse than the best model but 6.27% (3.89 percentage points) better than the base model.

#### External validation

Figure 15 plots the number of excluded training subjects dependent on the mean accuracies achieved for the external AIBL data set. Tables 11 and 12 summarize those results. The no information rate for the AIBL data set was 57.14%, and the base model reached a mean accuracy of 60.00%, which was slightly worse than the results achieved for RF models presented in Fig. 9. The RF Data Shapley method showed some improvements in the classification results. The best accuracy of this model was 66.43% achieved by excluding 195 subjects. The validation cutoff of this strategy was 133, and the associated models reached a mean classification accuracy of 61.79%, which was 2.98% (1.79 percentage points) better than the base model.

The course of the LR Data Shapley method was more conspicuous, as the accuracies slightly increased until approximately 75 subjects were excluded from the training set. After this cutoff value, the method shows a rather constant course until approximately 300 training subjects were excluded. After this threshold, the accuracies of this method increased until a peak of 75.36% was reached by excluding 396 subjects and thus 84.80% of the training set. However, the small number of subjects included in these models increased the risk of a selection bias. The validation cutoff value of this method was 248, and the associated model reached a mean accuracy of 58.57%, which was 2.38% (1.43 percentage points) worse than the base model.

#### Interpretation model

**Fig. 16:**
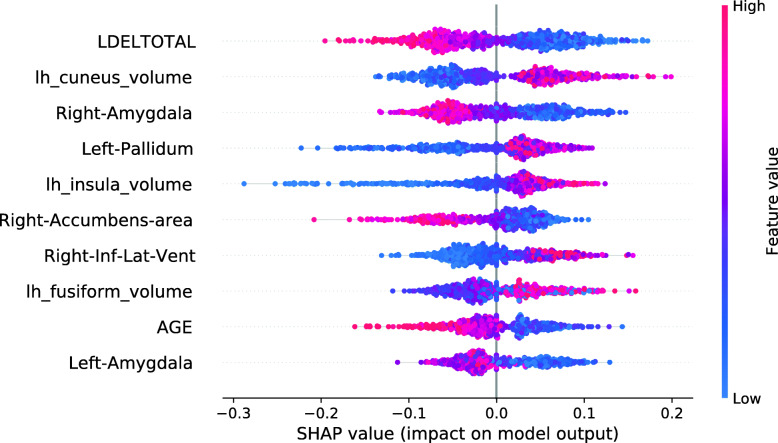
SHAP summary plot for the XGBoost model. No training subjects were excluded. SHAP summary plots aggregate the explanation of individual predictions of the entire training and test set. Each point visualizes a Shapley value for a subject and a feature. The color of the points depends on the feature values, and the horizontal axis shows the calculated Shapley values. The vertical axis represents both the features, ordered by the mean absolute Shapley values and their distribution. The positive class is pMCI

Figure 16 shows the SHAP summary plot for an XGBoost base model. The most important feature in this model was the LDELTOTAL cognitive test score. The model learned those poor test performances were associated with disease progression, whereas good test performances had a protective effect. The second most important feature in this plot was the volume of the left cuneus. Strikingly, high volumes of the left cuneus were associated with AD conversion. As this model partly associated high brain volumes with disease progression, some associations were not biologically plausible [61, 65–67]. These features were the volumes of the left cuneus, the left pallidum, the left insula, and the left fusiform gyrus.

**Fig. 17:**
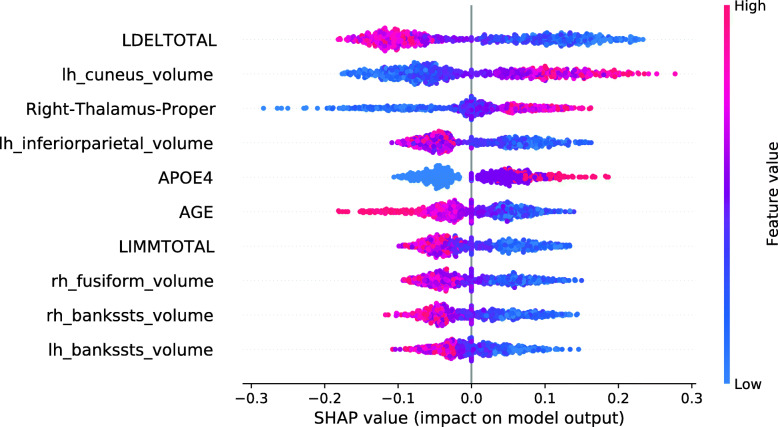
SHAP summary plot for the XGBoost model. 248 training subjects with the smallest LR Data Shapley values were excluded. SHAP summary plots aggregate the explanation of individual predictions of the entire training and test set. Each point visualizes a Shapley value for a subject and a feature. The color of the points depends on the feature values, and the horizontal axis shows the calculated Shapley values. The vertical axis represents both the features, ordered by the mean absolute Shapley values and their distribution. The positive class is pMCI

Figure 17 shows the SHAP summary plot for an XGBoost model trained on the entire training set, except for those 248 subjects with the smallest LR Data Shapley values. The cutoff value of 248 was the validation cutoff value for the LR Data Shapley method. Consistently with the previously described base model, the most important feature in this model was the LDELTOTAL cognitive test score. The model learned that poor test performances were associated with disease progression, whereas high LDELTOTAL cognitive test scores were associated with a stable MCI diagnosis. The second most important feature was the left cuneus volume. For this feature, the model learned an association that was not biologically plausible. The same was observed for the right thalamus proper volume, which was the third most important feature in this plot. However, the number of features with an implausible association was decreased in comparison to the base model. It can be also observed that the number of ApoE *ε*4 alleles was more relevant in the model with the reduced data set. Overall, the SHAP summary plots mainly showed less complex ML models for the reduced training sets.

## Discussion

In this research, an ML workflow was developed to distinguish between sMCI and pMCI subjects. Data used in the experiments included non-invasive MRI, cognitive test scores, and demographic data from two AD cohorts. Data Shapley values were used to avoid overfitting of the ML models and thus focus on the most important AD patterns. Data Shapley values were successfully applied to other medical contexts, such as in pneumonia detection [37] in the Chest X-Ray data set [38]. Therefore, in this research, Data Shapley values were applied to AD data sets. The experiments performed in this article showed slightly improved classification results achieved when excluding training subjects using TMC Data Shapley in comparison to the random exclusion and the LOO methods. The results of the RF Data Shapley exclusion showed slightly better results for the independent ADNI test set. RF and XGBoost classifiers were used for the final classification of sMCI and pMCI subjects, and both models showed similar classification performances. However, the XGBoost models showed better improvements using Data Shapley.

For all experiments, except for the RF models and the AIBL test set, the Data Shapley outperformed all the other methods comparing the results of the validation cutoffs. For the cutoff values which were determined for the test set, the Data Shapley method also outperformed the LOO and random exclusion methods. However, those results were not tested for an independent test set and thus might be an optimistic estimation.

The models trained exclusively on subjects with positive Data Shapley values (zero-cutoff models) often reached promising classification results.

The used feature selection method selected features, which were previously associated with AD progression.

The associations between the Data Shapley values and the model features were investigated using SHAP summary plots within the diagnostic groups. Complex relations were observed between the Data Shapley values and the features. The most important features to predict the LR Data Shapley values in the sMCI diagnostic group were the number of ApoE *ε*4 alleles and the left inferior parietal lobule. The most important features in the pMCI group were the volume of the right thalamus proper and the LDELTOTAL cognitive test score. The most important features to predict the RF Data Shapley values in the sMCI group were the LDELTOTAL cognitive test score and the volume of the left amygdala. The LDELTOTAL cognitive test score and the volume of the left supramarginal gyrus were the most important features to predict the RF Data Shapley values in the pMCI group. Most of the associations noted, were biologically plausible, as small brain volumes, bad cognitive test performance and presence of ApoE *ε*4 alleles were more representative for pMCI subjects. It is important to note that the Data Shapley method increased the risk of a selection bias for the models trained on reduced data sets.

Previous studies in AD detection especially those, which used deep learning models, suffered from data leakage [7]. The proposed ML workflow was carefully validated for two independent data sets. First, the model selection was performed using an independent validation set. The final models were validated using an independent ADNI test set, which included no training or validation subjects. Additionally, an external data set from the AIBL cohort was used to validate the results.

Many other ML models for AD detection were trained and validated for a single data set [75] and thus lacked for external validation. External validation is important in ML [76] because most AD cohorts differed regarding study locations, study size, recruitment criteria, diagnosis method, and biomarkers. [77–80].

The experiments presented in this research showed the generalizability of the ML models for the AIBL data set. The Data Shapley valuation strategy showed small improvements for both base models. However, the AIBL accuracies achieved for the validation cutoff values often achieved results similar to the base models.

Another disadvantage of AD detection models was the poor reproducibility [75] of the results. Although most of the ML models for early AD detection were trained on the ADNI data set [75], they use different subject selections to train and validate their models. Some benchmark challenges, for example, The Alzheimer’s Disease Prediction Of Longitudinal Evolution (TADPOLE) [81], and the Computer-Aided Diagnosis of Dementia (CADDementia) [82] challenge, provided fixed data sets. However, this leads to less flexibility and no consideration of new observations. A framework, which includes standardized pipelines for subject selection, preprocessing, feature extraction, classification algorithms, and cross-validation, was developed in Samper-Gonzáles et al. [75]. Data leakage problems additionally hindered the comparability between ML models in AD detection [7].

In this article, reproducibility was addressed, providing a precise description of the data set and the inclusion and exclusion criteria. At the time of publication, a software tool that enables the generation and description of reproducible AD data sets will be published.

Many articles trained black-box models for AD detection without providing a model interpretation. The interpretation of ML models is important in healthcare [83] to trust complex models.

There have been already some articles proposing interpretation methods for black-box models in AD detection. Most of those articles showed promising results [39, 79, 84].

Here, Kernel SHAP values were used to interpret black-box models. In this context, SHAP summary plots were used to examine if the trained models show biologically plausible relations. In the experiments, only a few implausible relationships were identified. Models which were trained on reduced data sets, showed biologically plausible associations. However, the influence of the number of ApoE *ε*4 alleles and the LDELTOTAL cognitive test score was much more present in those models. The models trained on the reduced data sets showed a decreased number of biologically implausible associations. These results support the assumption that Data Shapley valuation can help to avoid model overfitting but might also facilitate a selection bias. Thus, future work will address this issue using larger data sets. SHAP force plots were used to interpret individual diagnoses. The explanation of individual model decisions and the associated possibility to model the influence of single feature expressions make Shapley values more valuable in comparison to classical feature importance measurements.

### Limitations

The approach proposed in this article had several limitations. First, the Data Shapley method increased the possibility of a selection bias, which leads to more specific and less generalizable models and thus reduced the problem to a specific subgroup. For this reason, it is important to reproduce the results described in this paper on a larger AD data set. Thus, the validation set used to calculate Data Shapley values would include more diverse MCI subtypes. The small number of subjects in this research results from the small number of MCI subjects with a longitudinal diagnosis available in the ADNI cohort. Additionally, the AIBL data set focuses on CN subjects, and thus the external validation set included only 28 subjects. Future investigations should thus include more AD data sets, knowing that those cohorts differ in their inclusion criteria. The AD subset [85] of the Heinz Nixdorf Risk Factors Evaluation of Coronary Calcification and Lifestyle (RECALL) (HNR) [86], the Open Access Series of Imaging Studies (OASIS) [87], or a subset of the National Alzheimer’s Coordinating Center (NACC) [88] can be used as supplementary cohorts. Another fact that lead to a small number of training samples is that the LOO and Data Shapley valuation strategies need an independent validation data set. Future work will use bootstrapping as a wrapper function to overcome this limitation.

Due to the consistent availability in the examined data sets, only MRI, demographics, the number of ApoE *ε*4 allele, and cognitive test data are included in our investigations. However, PET scans and biomarkers have high medical relevance and should thus be considered in future investigations.

The results of the experiments included both maximum exclusion cutoffs for the validation set and the test set. However, it should be noted that the exclusion cutoffs for the test data set performed optimistic estimations not validated with an additionally independent validation set.

Another limitation was that no hyperparameter-tuning was performed within the workflow. It was expected that adding this process would increase the computational effort of the workflow and impede the interpretation of the results. It would also require another independent validation set, which would not be feasible as the data is already sparse.

In this work, only classifiers, based on decision trees, were used to distinguish between sMCI and pMCI subjects. Although only decision tree-based classifiers were used, a model-agnostic interpretation method was chosen to simplify the extension to other ML models. It is interesting to investigate the presented method for other ML models. In particular, deep learning models like CNNs, which were can automatically extract locally textural features from MRI scans, were often used to improve classical ML methods. However, there is no consensus if those methods can improve AD detection. Most of the previous work in this area suffered from data leakage [7] or investigated the less challenging discrimination between AD and CN.

## Conclusion

This work proposed an ML workflow to identify whether MCI subjects will prospectively convert to AD. Differentiation of these is important to recruit and monitor subjects for therapy studies. The data used in this approach were non-invasive and included MRI scans, demographic data, the number of ApoE *ε*4 alleles, and cognitive test results. Volumetric features were extracted from the MRI scans using the FreeSurfer pipeline. Data used in the experiments were received from two cohorts: ADNI and AIBL. All models were trained on a training set of the ADNI cohort and validated for two independent test sets and additionally by an independent validation set. On the one hand, an independent test set of the ADNI cohort and a subject selection from the AIBL cohort. In particular, it was examined whether Data Shapley values were able to identify the subjects with the most informative data and thus improve the classification results of the trained models. Data Shapley values were computed for the independent validation set. RF and XGBoost models were trained and interpreted using Kernel SHAP.

The results of the experiments showed improvements for the independent test set through the used TMC Data Shapley method. The SHAP summary plots mainly showed biologically plausible associations for both RF and XGBoost models. Less complex models, focusing on the ApoE *ε*4 alleles and cognitive test results, were learned if training subjects with small Data Shapley values were excluded. The results for the independent AIBL data set showed reproducible results.

## Appendix

### Shapley values

Shapley values [36] are affiliated with coalition game theory. The aim is to fairly determine the effect of every single player on the overall team result. It is assumed that *n* players play a cooperative game. The outcome of the game is referred to as *V*(*D*), where *D*={1,...,*n*} denotes the aggregated set of players. *Φ* is the contributed value of each player to the outcome of the game. An intuitive method is the LOO method, in which the game is first played with all players, and then with the entire set of players but without the player at interest *i*. It can be seen in Eq. 8 that the value of each player is the difference between the game result with the entire data set minus the game result without the player at interest.

8 $$ {\Phi_{i}=V(D) -V\left(D\setminus\{i\}\right)}  $$

To fairly distribute the values of all players, the sum of all individual values *Φ*_*i*_ is required to correspond to the overall result of the team, which can be seen in Eq. 9. The LOO method does not meet this criterion.

9 $$ V(D)=\sum_{i=1}^{n} \Phi_{i}  $$

The Shapley values offer an alternative approach, which fulfills this criterion. To fairly distribute the values of the players, each Shapley value considers all subsets *S* of players. The weighted sum of the individual performances in the subsets then gives the player’s overall individual performance. Shapley values are thus defined according to Eq. 10.

10 $$ {\Phi_{i}=\sum_{S\subseteq D\setminus\{i\}}\frac{V\bigl(S\cup\{i\}\bigr)-V(S)}{\dbinom{n-1}{|S|}}}  $$

**Table 13:** Parameters used for the implementation of the ML workflow

Method	Hyperparameter	Values
RF	n_estimators	50
	criterion	“gini”
	max_depth	None
	min_weight_fraction_leaf	0.0
	max_features	“auto”
	max_leaf_nodes	None
	min_impurity_decrease	0.0
	min_impurity_split	None
	bootstrap	True
	oob_score	False
	class_weight	None
	ccp_alpha	0.0
	max_samples	None
XGBoost	subsample	0.6
	objective	“binary:logistic”
	booster	“gbtree”
	eta	0.3
	gamma	0
	max_depth	6
	min_child_weight	1
	max_delta_step	0
	sampling_method	“uniform”
	colsample_bytree	1
	colsample_bylevel	1
	colsample_bynode	1
	lambda	1
	alpha	0
	tree_method	“auto”
	sketch_eps	0.03
	scale_pos_weight	1
	updater	“grow_colmaker,prune”
	refresh_leaf	1
	process_type	“default”
	grow_policy	“depthwise”
	max_leaves	0
	max_bin	256
	predictor	“auto”
	num_parallel_tree	1
LR	solver	“liblinear”
	penalty	“l2”
	dual	False
	tol	1e-4
	C	1.0
	fit_intercept	True
	intercept_scaling	1
	class_weight	None
	max_iter	5000
	multi_class	“auto”
	warm_start	False
	l1_ratio	None
Data Shapley	number of repetitions	4
	model_family	{“RandomForest”, “logistic”}
	metric	“accuracy”
	num_test	108
	problem	“classification”
	sample weights	None
	save_every	100
	err	0.1
	tolerance	0.01
	g_run	False
	loo_run	True
Kernel SHAP	nsample	3000
	l1_reg	“auto”
	link	“identity”

### Hyperparameters of implementation

## Data Availability

De-identified data used in preparation of this article were obtained from the Alzheimer’s Disease Neuroimaging Initiative (ADNI) (https://adni.loni.usc.edu, Accessed: 18 May 2021) and the Australian Imaging, Biomarker and Lifestyle Flagship Study of Ageing (AIBL) database (https://aibl.csiro.au/, Accessed: 18 May 2021). Details about data access are detailed there. The authors had no special access privileges others would not have to the data obtained from the Alzheimer’s Disease Neuroimaging Initiative (ADNI) and the Australian Imaging, Biomarker and Lifestyle Flagship Study of Ageing (AIBL) database.

## Abbreviations

MCI: Mild cognitive impairment
AD: Alzheimer’s disease
ML: Machine learning
MRI: Magnetic resonance imaging
ADNI: Alzheimer’s Disease Neuroimaging Initiative
AIBL: Australian Imaging
Biomarker and Lifestyle Flagship Study of Ageing; RF: Random forest
XGBoost: eXtreme Gradient Boosting
SHAP: SHapley Additive exPlanations
LR: Logistic regression
pMCI: Progressive mild cognitive impairment
sMCI: Stable mild cognitive impairment
DBSCAN: Density-based spatial clustering of applications with noise
LOF: Local outlier factor
GAN: Generative Adversarial Network
SSD: Self-Supervised outlier detection
CN: Cognitive normal
LDA: Linear discriminant analysis
GM: Grey matter
ROI: Region Of interest
FDG: Fluorodeoxyglucose
PET: Positron emission tomography
SNP: Single-nucleotide polymorphism
SVM: Support vector machine
CNN: Convolutional neural network
ISM: Instances that Should be Misclassified
PRISM: PReprocessing Instances that Should be Misclassified
UCI: University of California
Irvine; NINCDS-ADRDA: National Institute of Neurological and Communicative Disorders and Stroke–Alzheimer’s Disease and Related Disorders Association
BL: Baseline
ApoE *ε*4: ApolipoproteinE *𝜖*4
MMSE: Mini-Mental State Examination
CDR: Clinical Dementia Rating
DKT: Desikan–Killiany–Tourville
eTIV: Estimated total intracranial volume
LOO: Leave-one-out
TMC: Truncated Monte Carlo
ACC: Accuracy
F1: F1-Score
LIME: Local Interpretable Model-agnostic Explanations
LSTM: Long short-term memory
RNN: Recurrent neural network
RECALL: Risk Factors Evaluation of Coronary Calcification and Lifestyle
HNR: Heinz Nixdorf Risk Factors Evaluation of Coronary Calcification and Lifestyle
FCN: Fully convolutional network
MLP: Multilayer perceptron
FHS: Framingham Heart Study
NACC: National Alzheimer’s Coordinating Center
t-SNE: t-distributed stochastic neighbour embedding
TADPOLE: The Alzheimer’s Disease Prediction Of Longitudinal Evolution
CADDementia: Computer-Aided Diagnosis of Dementia
OASIS: Open Access Series of Imaging Studies
DT: Decision tree
Grad-CAM: Gradient-weighted Class Activation Mapping
